# Importance and Potential of Rare Disease Research in Pediatric Rheumatology and Beyond: Pushing Frontiers

**DOI:** 10.1002/acr2.70138

**Published:** 2025-11-26

**Authors:** Christian M. Hedrich

**Affiliations:** ^1^ Department of Women's and Children's Health, Institute of Life Course and Medical Sciences, University of Liverpool and Department of Paediatric Rheumatology Alder Hey Children's NHS Foundation Trust Liverpool United Kingdom

## Abstract

Although individually occurring in less than 1 in 2,000 people, cumulatively, more than 7,000 rare diseases affect approximately 6% of the population worldwide. Children and young people are disproportionally challenged in number and severity, which may be explained by the large proportion of genetic conditions among rare diseases (70%–80%). Indeed, an estimated 30% of children with rare diseases do not survive past their fifth birthday. Because rare diseases are frequently missed or diagnosed with a delay of several years and <5% of rare diseases have a licensed treatment, the impact of rare diseases on the indivual affected (independent of age) and wider society is significant. To address these challenges sufficiently, rare disease expert centers combining research activity with patient care are needed to develop diagnostic tests, prognostic tools, and new treatments. This expert‐driven approach promises expedited diagnosis and efficacious treatment and care. Although restricted by chronic underfunding, rare disease research keeps delivering new exciting treatment options and technologies, some of which have revolutionized care not only in niche areas of medicine but also common diseases (the use of interleukin‐1 blockers in gout or COVID‐19–associated hyperinflammation, etc). However, rare disease research and care will only be successful in collaborative, mutidisciplinary and multiprofessional teams that involve patients and families as equal partners and span across institutional and national borders. Lastly, the use of state‐of‐the‐art computational approaches to share knowledge and associate molecular with clinical phenotypes, treatment responses, and disease outcomes will amplify our ability to serve patients and the society.

## Introduction

Following the definition from the World Health Organization (https://apps.who.int/gb/ebwha/pdf_files/EB156/B156_CONF2-en.pdf), rare diseases affect fewer than 1 in 2,000 people. In the United States, rare diseases are defined in the US Orphan Drug Act (1983) as health conditions affecting <200,000 patients in the United States.^1^ Nonetheless, because >7,000 individual rare diseases exist, 1 in 17 people (6% of the global population) will experience a rare disease during their lifetime.^2^ This translates to >400 million people affected by rare diseases worldwide and >3.5 million in the United Kingdom alone.^3^, ^4^

The importance of focusing on rare disease research and care programs becomes especially evident in children who are disproportionally affected. Approximately 75% of rare diseases manifest during childhood, and 50% of patients with rare diseases are children, a group representing only 20% to 25% of the population.^2^, ^5^ The impact of rare diseases on children is further amplified by their severity and associated unfavorable outcomes. Notably, a substantial proportion of children affected (estimated 30%) do not survive past their fifth birthday.^2^ The early onset and severe phenotype of many rare diseases may be explained by their genetic origin (approximately 70%–80%).^6^

In misalignment with patient needs, rare disease research and care remains underfunded.^7^ This is particularly evident when considering child health research (covering approximately 20%–25% of the population in Western countries and 50% of all patients with rare diseases^2^, ^5^). Although exact numbers are difficult to generate, in the United Kingdom, an estimated 6% of National Institute of Health Research (NIHR) funding is awarded for pediatric research, and the US National Institutes of Health (NIH), between 2011 and 2020, awarded 9.4% of their grants for pediatric research.^8^ This contributes to delayed knowledge progression across child health and rare disease programs, a limited number of specialized centers, and accrual of damage and disability of patients affected.^4^, ^9^, ^10^ Proportionate funding of pediatric programs (serving 20%–25% of the general population) would likely almost triple research funds for child‐focused research (including 50% of patients with rare diseases across age groups and 75% of all rare diseases^2^). The associated likely benefits are supported by statistics. On average, it takes five years to diagnose patients with rare diseases, and frequently, no treatments are available. Indeed, <25% of rare diseases have a known molecular cause, and 95% of rare diseases lack approved treatments.^2^ Meanwhile, rare diseases also pose a significant financial burden on health care services internationally. Data from Australia suggest that 0.5% of the population experience single chromosome disorders but cause 1.9% of hospital admissions and 1.5% of hospital costs.^3^ This does not even include societal cost and impact on an individual's wellbeing and ability to generate income.

Undoubtedly, a better mechanistic understanding and resulting diagnostic and treatment approaches will improve health and wellbeing and reduce the cost for rare disease populations and society. Although rare diseases are, in most cases, caused by defects in single (monogenic) or a small number of genes (oligogenic), more common diseases most frequently are the result of complex dysregulations affecting several genes or pathways.^7^ However, genetically complex contributors to common diseases frequently overlap with, and are therefore informed by, rare diseases with shared clinical features (examples include rare monogenic interferonopathies and classic systemic lupus erythematosus (SLE),^11^ autoinflammatory chronic nonbacterial osteomyelitis and osteoporosis,^12^, ^13^ etc). Thus, rare disease research will also inform common diseases, thereby amplifying its impact on population health.^7^ However, claiming new frontiers, rare disease programs are facing several key challenges and opportunities.

### Pediatric rare disease research to improve diagnosis and population health

Children are not only disproportionally affected by rare diseases but also represent a perfect study population for many, including systemic inflammatory diseases.^6^, ^14^ Frequently, disease phenotypes are more severe in pediatric‐onset disease when compared with adult‐onset disease (especially in the field of systemic autoimmune and/or inflammatory disease). Children and young people usually exhibit reduced lifestyle and/or aging‐related health impact. They have fewer and/or no comorbidities and consume fewer medications, drugs, alcohol, etc, that may impact molecular and clinical phenotypes, thereby complicating research into disease mechanism, target identification (because of molecular background noise), and treatment.^6^, ^14^

In genetically complex diseases, such as SLE, rare gene variants with high functional impact are more common among children and young people as compared with patients with disease onset during adulthood, in which risk alleles in the presence of acquired molecular events (eg, epigenetic changes) may dominate.^11^, ^14^ Notably, rare damaging gene variants in many cases promote a better understanding of genes and their involvement in molecular pathways and body functions. Indeed, damaging variants affecting the immune system may be seen as the equivalent to knockout or forced expression models.^6^, ^14^ Collaborative lifespan approaches promise an improved understanding of genotypic variation across age group in some rare diseases, such as in SLE, starting with damaging variants and associated pathways to define wider risk alleles that may have cumulative impact.^11^

Because environmental and lifestyle impacts on epigenetic signatures are strong and difficult to measure, pediatric rare disease research promises new insights into the epigenetic code and associated gene regulatory impact.^14^, ^15^ Several genetic diseases affect DNA methylation patterns (eg, Rett syndrome, methyl‐CpG binding protein 2 variants), resulting in altered tissue differentiation and/or function, sometimes beyond the originally described phenotype,^16^, ^17^ whereas imprinting disorders are characterized by impaired epigenetic silencing of genes (eg, Prader‐Willi, Angelman, and Beckwith‐Wiedemann syndromes) in the absence of known underlying genetic causes.^18^

Understanding genetic and epigenetic contributors to rare diseases will improve not only diagnostic approaches but also risk assessment, phenotype, and outcome prediction. Because complications of rare diseases (such as cardiovascular disease, metabolic syndrome, etc) also affect large proportion of the otherwise healthy population, knowledge from rare disease research will likely benefit the wider population and improve health outcomes.^19^, ^20^

### Rare diseases as a public health challenge

The importance of patient and family involvement in research is even more evident in rare disease when compared with common diseases.^10^ Working with patients and families and advocacy groups facilitates learning, amplifies research impact, and can apply pressure on regulatory bodies to implement recent knowledge into routine care, especially during financially challenging times.^3^ The observation that rare diseases, especially genetically mediated ones, more frequently affect minority ancestral groups, alongside the fact that disease impact is particularly high in socioeconomically challenged populations, underscores the importance of making patient and family involvement accessible to all societal groups.^9^, ^21^

The rarity of diseases makes national and international partnerships necessary to share knowledge, data, research expertise, and analytic tools.^3^ Based on pronounced differences between health care and wider political systems, significant differences exist internationally. Some countries have government‐funded research institutes that deliver state‐of‐the‐art research and care, such as the NIH in the United States (NIH Undiagnosed Diseases Program^22^ and NIH Undiagnosed Diseases Network^23^) or the German Centres for Rare Diseases (https://www.se-atlas.de/?ln=en_EN).^24^ In the United Kingdom, recently established Rare Disease Research UK nodes, jointly funded by the Medical Research Council and the NIHR, are steps in the same direction.^25^ Others established rare disease networks on a continental level, such as the European Research Network (ERN) (https://health.ec.europa.eu/rare‐diseases‐and‐european‐reference‐networks/european‐reference‐networks_en) for Rare Immunodeficiency, Autoinflammatory and Autoimmune Diseases (RITA) or the Undiagnosed Diseases Network International.^21^ Shared features of all these programs include their collaborative nature characterized by the generation of high experiences across multiprofessional and multidisciplinary teams, harmonization of diagnostic and treatment algorithms, evidence generation, and international sharing of knowledge. In these regards, rare disease programs even have a political mission in times in which nationalist beliefs and isolationist ideologies re‐emerge, driving national politics. In such wider interdisciplinary and interprofessional team approaches, developing expertise in a group of diseases or therapies (as opposed to low expertise levels across all) will amplify knowledge gain and patient benefit.

Rapidly developing computational and artificial intelligence–driven approaches are also of growing importance in the rare diseases field and promise amplified knowledge gain through machine learning and pattern recognition. Several data sharing platforms are already available (eg, GeneMatcher,^26^ Matchmaker Exchange,^27^ and MyGene2^28^) to identify centers caring for patients with similar phenotypes and/or genotypes.^3^ However, more complex computational platforms are in the making to link phenotypes with molecular signatures, treatment responses, and disease outcomes (“VERY big data”). Linking centers caring for patients with rare diseases will amplify our ability to improve diagnosis, care, and associated outcomes.^29^

Because approximately 75% of rare diseases manifest during childhood and are associated with high morbidity and mortality rates, adult‐focused providers may lack experience and confidence with (at least) some of them.^2^ This can complicate the transition from pediatric to adult care, which is an ongoing challenge even for patients with more common diseases.^30^ Thus, team approaches and establishing rare disease centers, covering the age spectrum, is essential to improve care and outcomes.^31^ Much may be learned from existing transition programs, as in rheumatology, for example.^32^ Indeed, the ERN for RITA has demonstrated that harmonized diagnostic and therapeutic approaches, data collection and exchange, and the establishment of structured transition programs to adult care improve patient experience and disease outcomes.^33^, ^34^

### Development of state‐of‐the art treatments

Currently, only approximately 5% of rare diseases have approved treatments, which links not only to the complexity of rare diseases but also the small number of patients experiencing a single defined rare disease, limiting financial gain for the pharmaceutical industry.^2^, ^3^, ^4^ However, several medicines developed and approved for rare diseases have subsequently been used in and (sometimes) approved for use in more common conditions.^35^ Examples include the use of interleukin‐1 (IL‐1) blockers in gout,^35^ hyperinflammatory responses to SARS‐CoV‐2 in children (postacute pediatric inflammatory multisystem syndrome or multisystem inflammatory syndrome in children),^36^, ^37^ or JAK inhibitors in the care for patients with acute COVID‐19 or chronic long‐term complications.^38^

Over recent years, gene therapy has been introduced into the care of several rare disease areas. Learning from complications associated with early approaches (secondary malignancies, liver toxicity and neurotoxicity, etc), replacement of defective genes using viral or nonviral vectors has been safely introduced into the care of patients with rare diseases, such as spinal muscular atrophy, inborn errors of immunity and metabolism, hemoglobinopathies, and inherited blindness.^39^ Delivery of messenger RNA (mRNA) may be applied as a future (at least temporary) alternative to vector‐mediated gene delivery. Indeed, in preliminary animal studies, mRNA technology has been used to replace enzymes,^40^ which promises potential also for its use in human disease (such as hyperinflammatory mevalonate kinase deficiency, also known as hyperimmunoglobulinemia D with periodic fever syndrome (HIDS)^41^ or DNase 1‐like 3 (DNase1L3) deficiency,^6^ etc).^42^ In addition to the recently celebrated application of mRNA technology to vaccinate against SARS‐CoV‐2, it can be applied to train a patient's immune system to recognize and attack cancer cells expressing neoantigens, promising potential in personalized treatment approaches.^42^ Similar to current approaches targeting malignancies, future applications in systemic autoimmune disease may be developed to deplete distinct immune cell subpopulations expressing disease‐associated surface proteins.^43^

An alternative to gene delivery may be gene editing based on CRISPR/Cas technology. Using novel technology, individual nucleotides or small sequences can be precisely targeted and replaced, promising significant therapeutic value in genetically mediated diseases.^44^ While CRISPR/Cas technology has widely been used to study disease in vitro and in vivo (in animals), over 20 clinical applications, including sickle cell disease and β‐thalassemia, have been reported.^44^ Indeed, a most recent report on the use of in vivo genome editing through lipid nanoparticle–based delivery in a patient with carbamoyl‐phosphate synthetase 1 deficiency suggests wider applications of this technology in the future.^45^ Although long‐term follow‐up is required to assess safety and efficacy, this approach promises significant therapeutic potential across genetically mediated diseases with limited side effects (as compared with, eg, conditioning before stem cell transplantation) and reduced cost.^45^

Epigenetic engineering aims to correct disease‐associated epigenetic alterations. In cancer, epigenetic treatments have been introduced into routine care, including untargeted histone deacetylase inhibitors (vorinostat and romidepsin)^46^ and DNA methyltransferase inhibitors (azacytidine and decitabine).^47^ However, although valuable in lethal cancers, genome‐wide epigenetic changes mediated by currently available drugs may not be suitable for all diseases. Epigenetic marks in, for example, SLE are region and context specific and may be bidirectional,^14^, ^15^, ^48^ and targeted approaches are necessary for optimal effects and limited toxicity. Building on existing tools for CRISPR/Cas genome editing, epigenetic engineering promises the correction of disease‐associated epigenetic alterations in a region‐specific manner using guide RNAs (gRNAs) that direct the modified CRISPR/Cas system to regulatory regions around target genes. Notably, single gRNAs do not complex with orthologous Cas enzymes. Thus, combined delivery of inhibiting and activating constructs is possible (eg, up‐regulate one gene while repressing another).^14^, ^15^, ^18^, ^48^

Recently, oligonucleotide treatments have been introduced into rare disease therapy to control and/or correct gene expression. Antisense oligonucleotides bind to RNA sequences coding for proteins. Through this, they can correct reduced gene expression (eg, through splice‐modulation), suppress defective gene expression (knock‐down), or modify function of proteins.^49^, ^50^ RNA interference is a physiologic process preventing RNA transcription or its translation into protein. This can be used therapeutically to induce sequence‐specific inhibition of target gene expression or translation. Small‐interfering RNAs (siRNA) or microRNAs (21–22bp) can be delivered to cells in nanoparticles,^50^, ^51^ and siRNAs are already used in the treatment of polyneuropathy in people with hereditary transthyretin‐mediated amyloidosis.^52^

Beyond their established benefit in hematologic malignancies, chimeric antigen receptor (CAR) T cells have recently been introduced into the treatment of autoimmune and inflammatory diseases,^53^, ^54^, ^55^ including SLE in children.^56^ T cells are collected from the patient, genetically modified through introduction of a CAR (gene therapy), and expanded to then be infused back into the patient where they attack their target cells (tumor cells in cancers and B cells in SLE). This approach is highly effective in eliminating target cells but can be associated with significant side effects, such as cytokine release syndrome, immune effector cell–associated neurotoxicity syndrome, and/or immune effector cell–associated hemophagocytic lymphohistiocytosis–like syndrome, that require intensive care treatment.^57^ Although first reports suggest that treatment‐associated complications may occur less commonly and severely in autoimmune diseases when compared with cancers, data are preliminary, and published literature is, most likely, subject to reporting bias toward successful cases.^53^, ^54^, ^55^ Moreover, the significant cost for CAR T cell generation, conditioning, and transfusion treatment will likely reserve this approach for the most complex and treatment‐resistant cases.^53^, ^54^, ^55^ However, bispecific antibody treatment, linking target cells with T cells in vivo, so‐called bispecific T cell engagers, may offer a more affordable and well‐tolerated alternative for milder cases.^58^

Lastly, the identification of disease‐associated molecular alterations and pathway dysregulation offers ample opportunity for the development of new small‐molecule^59^ or antibody treatments^60^, ^61^ that may also be effective in more common diseases with related pathology. Because underlying technologies have been widely implemented in routine care, they are not discussed in detail here.

### Innovative and/or adaptive trial design for rare disease areas

Commonly used randomized controlled trial designs comparing medications with placebo are not suitable for rare diseases because the sample sizes necessary (sometimes hundreds of patients per group) are not achievable. Furthermore, in some disease areas (especially systemic inflammatory disease), off‐label treatments are used, and withholding treatments in a study cohort, replacing them with placebo, may be unethical. Thus, new trial designs should be applied in smaller cohorts, using pre‐existing prior knowledge and allowing for continuous updating of probabilities as new data emerge. Prior knowledge can be extracted from previous studies, cohort data, and/or expert opinions (generated in prior meetings). Bayesian studies compare standard of care to new treatment options in a head‐to‐head comparison. These approaches reduce time to completion and cost while allowing patients to receive treatment (without the risk of being randomized to a placebo group). Bayesian trials have been successfully used to study treatment responses to pediatric autoimmune uveitis^62^, ^63^ and juvenile idiopathic arthritis.^64^

Indeed, Bayesian trial design used in small pediatric head‐to‐head trials started to be accepted by pharmaceutical industry and wider health services and are now also used across the age spectrum and disease areas.^65^, ^66^, ^67^ This is, indeed, of major importance to the rare disease field, as it helps in overcoming regulatory uncertainty, especially in the pediatric cohort. Generation of expertise in treatment development, testing, and implementation is therefore a key benefit of specialized rare disease centers and/or networks.

## Conclusions

In conclusion, securing the funds necessary to deliver on disease research and care can be a challenging task, especially in financially desperate times. However, several rare disease programs have been launched to improve the situation for individual patients and the wider population. Although published data on their real‐world impact are still somewhat preliminary, rare disease initiatives across Western countries are beginning to demonstrate their value through enhancing diagnoses and reducing cost through accumulation of expertise.^68^, ^69^, ^70^

Because rare diseases, although usually more severe, may share impaired signaling pathways and clinical features with common diseases, findings from rare diseases research have significant potential to benefit wider patient populations. In addition to serving local communities, it is our moral responsibility to make treatment affordable and accessible to all patients, including those in lower income environments. Although current treatments for rare diseases are, in most cases, prohibitively expensive, cost may be reduced through their licensing in common disease areas and/or the expiration of patent protection. Disappointingly, in the here‐mentioned exemplary case of IL‐1–blocking agents, this hope has not materialized yet. A possible alternative may be the replacement of novel, highly effective targeted treatments with cheaper (already existing) alternatives with similar molecular effects.

## AUTHOR CONTRIBUTIONS

Dr Hedrich contributed to at least one of the following manuscript preparation roles: conceptualization AND/OR methodology, software, investigation, formal analysis, data curation, visualization, and validation AND drafting or reviewing/editing the final draft. As corresponding author, Dr Hedrich confirms that all authors have provided the final approval of the version to be published and takes responsibility for the affirmations regarding article submission (eg, not under consideration by another journal), the integrity of the data presented, and the statements regarding compliance with institutional review board/Declaration of Helsinki requirements.

## Supporting information


**Disclosure Form**:
